# Effects of Coffee Decaffeination on Beverage Bioactives, Antioxidant Activity, and Lipid Metabolism in Hyperlipidemic Rats

**DOI:** 10.1111/1750-3841.71340

**Published:** 2026-08-21

**Authors:** Adriene Ribeiro Lima, Jeniffer Ferreira de Miranda, Juliana Mesquita Freire, Paula Kujbida, Sheila Andrade Abrahão, Eulália Mendes, José Avelino Alves Andrade da Silva, Fernanda Borges de Araújo Paula, Stella Maris da Silveira Duarte, Rosemary Gualberto Fonseca Alvarenga Pereira, Susana Casal

**Affiliations:** ^1^ Department of Bromatology Graduate Program in Applied Sciences to Health Products (PPG‐CAPS) Faculty of Pharmacy Fluminense Federal University (UFF) Niterói Rio de Janeiro Brazil; ^2^ Department of Chemistry Federal University of Lavras (UFLA) Lavras Minas Gerais Brazil; ^3^ Department of Pharmaceutical Sciences and Administration Faculty of Pharmacy Fluminense Federal University (UFF) Niterói Rio de Janeiro Brazil; ^4^ Bom Jesus do Itabapoana Campus Instituto Federal Fluminense (IFF) Bom Jesus do Itabapoana Rio de Janeiro Brazil; ^5^ LAQV–REQUIMTE, Faculty of Pharmacy University of Porto Porto Portugal; ^6^ Department of Chemical and Biological Engineering Faculty of Engineering University of Porto Porto Portugal; ^7^ Department of Clinical and Toxicological Analysis (DACT/FCF) Federal University of Alfenas (UNIFAL‐MG) Alfenas Minas Gerais Brazil; ^8^ Department of Food Science Federal University of Lavras (UFLA) Lavras Brazil

## Abstract

The aim of this study was to evaluate the effect of chemical decaffeination on two coffee species (*Coffea arabica* and *Coffea canephora*) in terms of the chemical composition of filtered coffee beverages and their influence on lipid profile and oxidative stress in rats with diet‐induced hyperlipidemia. Rats (*n* = 30) were divided into six groups: a standard diet control and a hypercholesterolemic diet control (both on water) and four hypercholesterolemic diet treatment groups receiving whole or decaffeinated *C. arabica* or *C. canephora* beverages for 42 days (7.2 mL/kg/day). Lipid profile and oxidative stress biomarkers were determined in blood and liver samples. Decaffeination significantly altered the bioactive composition of the beverages, particularly the levels of chlorogenic acids, trigonelline, and diterpenes. No significant differences in the biological outcomes were observed among the four coffee‐treated groups. Therefore, based on pooled means across coffee‐treated groups, coffee consumption reduced non‐HDL cholesterol from 94.0 ± 7.9 mg/dL in the hyperlipidemic control group to 72.0 ± 11.4 mg/dL (23.5% reduction), triacylglycerol from 80.0 ± 8.8 to 44.6 ± 5.4 mg/dL (44.3% reduction), hepatic lipid content from 10.8 ± 0.8% to 8.1 ± 0.8% (25.2% reduction), and hepatic lipid peroxidation markers from 85.7 ± 7.7 to 28.0 ± 4.6 nmol MDA/mg protein (67.3% reduction). Overall, the hypolipidemic and antioxidant effects of coffee were independent of species and caffeine content. These findings provide insights into the impact of dichloromethane‐based decaffeination on the bioactive composition and biological effects of coffee beverages.

**Practical Applications:**

In a rat model of hyperlipidemia, decaffeinated coffee showed effects comparable to whole coffee on markers related to lipid metabolism and antioxidant protection. These findings may support the development and consumption of decaffeinated coffee products for individuals seeking to reduce caffeine intake.

## 1 Introduction

Coffee ranks among the most widely consumed beverages globally, appealing to consumers due to its distinct aroma, flavor, and stimulating effects, largely attributed to caffeine and other bioactive compounds (Klikarová et al. 2022). Annual coffee consumption is estimated at approximately 500 billion cups (Freitas et al. 2023).

Growing evidence indicates that moderate coffee intake may benefit human health. Consumption has been associated with reduced production of reactive oxygen species (ROS) and oxidative damage, potentially lowering the risk of cardiovascular, hepatic, metabolic, and certain types of cancer (Brandt et al. 2019; Li et al. 2023a). In addition, coffee intake has been linked to improvements in lipid profile, including reductions in low‐density lipoprotein (LDL‐c), total cholesterol, and triglycerides (El‐Kersh et al. 2023).

Two main commercial species of coffee are cultivated and traded, *Coffea arabica* (arabica coffee) and *Coffea canephora* (canephora coffee, including Conilon and Robusta varieties). These species differ in bean morphology and chemical composition, influencing the extraction of bioactive compounds during beverage preparation (Martins et al. 2023). Nevertheless, regardless of species, coffee contains a broad spectrum of bioactive compounds, such as phenolic compounds (e.g., chlorogenic acids and hydroxycinnamic acids), diterpenes, melanoidins, and caffeine (Gu et al. 2022). Caffeine, one of the best‐known bioactive compounds in coffee, is associated with increased alertness and attention, but excessive intake may cause adverse effects such as insomnia, headache, and elevated blood pressure (Zou et al. 2022).

The antioxidant role of caffeine remains controversial. While some studies suggest that caffeine may act as an antioxidant capable of reducing oxidative stress (Vieira et al. 2020), others consider the evidence insufficient to classify it as a consistent antioxidant compound (Petrucci et al. 2018). Consequently, the antioxidant capacity of coffee has been largely attributed to non‐caffeine constituents, particularly chlorogenic acids (Brandt et al. 2019; Gu et al. 2022; Vieira et al. 2020), which may exert enhanced effects through synergistic interactions among the bioactive compounds present in the beverage (Martins et al. 2023).

Due to the adverse effects that excessive caffeine may cause, many consumers choose decaffeinated coffee. Decaffeination is a technologically demanding process carried out on green coffee beans prior to roasting and can be achieved by different technologies, including extraction with organic solvents (e.g., dichloromethane, chloroform, ethyl acetate, ethanol, or diethyl ether), the Swiss Water process, supercritical CO_2_ extraction, and more recently, ultrasound‐assisted extraction (Klikarová et al. 2022; Pietsch 2017; Chindapan et al. 2025). Although decaffeination processes are designed to selectively remove caffeine, they may also affect other coffee constituents. Previous studies have reported alterations in sugars, proteins, lipids, aroma precursors, and other bioactive compounds, depending on the decaffeination method employed (Pietsch 2017; Toci et al. 2006). Consequently, decaffeination may modify the chemical profile of coffee and potentially influence its sensory and biological properties.

Although many studies have investigated the chemical composition and antioxidant activity of whole coffee beverages, a notable gap remains regarding how decaffeination affects the composition of coffee beverages and their biological effects, particularly in relation to oxidative stress and hyperlipidemia. Therefore, this study aimed to assess the impact of decaffeination on the chemical composition of filtered coffee beverages prepared from two coffee species (*C. arabica* and *C. canephora*) and to investigate their effects on lipid metabolism and oxidative stress in rats with diet‐induced hyperlipidemia.

## 2 Materials and Methods

### 2.1 Coffee Samples

Whole coffee bean samples, along with their corresponding dichloromethane‐decaffeinated counterparts from both *Coffea arabica* L. and *Coffea canephora* Pierre, were supplied by COCAM (Catanduva, SP, Brazil). Five hundred grams of each coffee sample were roasted using a Probatino roaster (Leogap, Curitiba, PR, Brazil, 1 kg capacity) to a medium roast level, Agtron disk #55, according to the Specialty Coffee Association (SCA) Roast Classification Color System. The roasted beans were ground (Pinhalense electric mill, Espírito Santo do Pinhal, SP, Brazil, model ML‐1) to a particle size of 20 mesh, packaged in polyethylene/aluminum containers, vacuum‐sealed and stored at ‐20°C until use.

#### 2.1.1 Preparing Coffee Beverages

Coffee beverages were prepared according to Nicoli et al. (1997), with modifications previously described by Silvério et al. (2013). Briefly, 10 g of ground coffee were placed in a commercial paper filter (No. 103), and 100 mL of deionized water at 90°C w‐ere poured over the coffee powder to obtain a conventional paper‐filtered coffee beverage. Aliquots (15 mL) of each coffee beverage were freeze‐dried and used as the starting material for all chemical analyses. For each determination, independent freeze‐dried aliquots were reconstituted and extracted according to the requirements of the respective analytical method. For the biological tests, fresh beverages were prepared at the time of administration to the animals.

### 2.2 Analysis of Bioactive Compounds in Coffee Beverages

#### 2.2.1 Determination of Total Phenolics

Total phenolics in the coffee beverages were estimated using the Folin–Ciocalteu colorimetric assay (Lima et al. 2010), with minor adaptations. Briefly, freeze‐dried coffee was reconstituted with deionized water to a final volume of 250 mL in a volumetric flask. An aliquot of 0.5 mL was transferred to a test tube with 2.5 mL of 10% (v/v) Folin–Ciocalteu reagent and 2 mL of 4% (m/v) sodium carbonate solution. Tubes were kept in the dark at room temperature for 2 h. Absorbance was then measured at 750 nm, and the results were expressed as g gallic acid equivalents/L.

#### 2.2.2 Determination of Caffeine, Chlorogenic Acids, Trigonelline, and Niacin Contents

Freeze‐dried coffee beverages were reconstituted with water (3 mL) and cleaned with Carrez I and II, plus methanol to remove polymeric components, and the resulting supernatant was analyzed by HPLC (Fujioka and Shibamoto 2008). Chromatographic separation was performed using a Jasco (Hachioji, Tokyo, Japan) HPLC system with a diode array detector (Jasco MD‐2015 Plus), using a reverse‐phase column (Phenomenex, Torrance, CA, USA; 250 × 4.60 mm; C18 ODS‐2; 5 µm) and the samples were eluted at 1 mL/min for 30 min at room temperature with a gradient of acetate buffer and methanol, following Chambel et al. (1997). Quantification was supported on individual standard calibrations with caffeine, 5‐caffeoylquinic acid (5‐CQA), trigonelline, and niacin. Identification of 3‐CQA and 4‐CQA isomers was based on their spectra and literature data and expressed as 5‐CQA equivalents, adjusted using the molar extinction coefficients of each isomer (Fujioka and Shibamoto 2008).

#### 2.2.3 Determination of Diterpenes: Kahweol and Cafestol

Freeze‐dried samples were reconstituted in hot water (5 mL) and subjected to cafestol and kahweol quantification according to Silva et al. (2012). Saponification was performed with 400 mg of KOH in a water bath (80°C) under agitation for 1 h. Diterpenes were extracted with 5 mL of diethyl ether and 2 mL of 2 M NaCl. The ether layer was separated, and the beverage was further extracted with four additional 5 mL portions of diethyl ether. The solvent was evaporated under a nitrogen stream, and the dried extract was reconstituted with the mobile phase. Separation was performed in a reverse‐phase column (Purospher STAR LichroCART, Merck Millipore, Darmstadt, Germany; 250 × 4 mm, 5 µm), using a water/acetonitrile mobile phase with a linear gradient (1 mL/min) for 35 min. Quantification was based on peak areas at 220 nm, based on standard calibrations of cafestol and kahweol.

#### 2.2.4 Determination of Tocopherols

Extraction was performed according to the methodology described by Alves et al. (2010). Freeze‐dried beverages were reconstituted with 5 mL of deionized water. BHT (1% in ethanol) and the internal standard tocol (2‐methyl‐2‐(4,8,12‐trimethyltridecyl)‐chroman‐6‐ol) were added, and extraction was carried out using methanol followed by liquid–liquid partition with n‐hexane/ethyl acetate (90:10, v/v). The organic phases were combined and evaporated under nitrogen, and the residue was redissolved in hexane prior to injection.

Separation was achieved by normal‐phase chromatography under isocratic elution using n‐hexane/1,4‐dioxane (99:1, v/v) on a Supelcosil LC‐SI column (Sigma–Aldrich, St. Louis, MO, USA; 7.5 cm × 3 mm; 3 µm) using a Jasco HPLC system equipped with a FP‐920 fluorescence detector. Tocopherols were identified by chromatographic comparison with authentic standards based on fluorescence detection (excitation at 290 nm and emission at 330 nm).

#### 2.2.5 Fatty Acid Composition

Freeze‐dried beverage samples were dissolved in methanol:dichloromethane (3:1, v/v) containing the internal standard (trinonadecanoate; tri‐C19) and left to react with 200 µL of acetyl chloride at 80°C for 1 h, then cooled, neutralized with potassium carbonate solution (K_2_CO_3_, 7%, w/v), and extracted twice with hexane. This extract was further cleaned with acetonitrile before evaporating to dryness under a nitrogen stream and reconstituted in dichloromethane.

Fatty acid methyl esters (FAMEs) were analyzed using a high‐resolution gas chromatograph (Chrompack CP‐9001, Chrompack, Middelburg, The Netherlands) equipped with a flame ionization detector (FID) and a split/splitless injection system. Separation was achieved on a fused silica capillary column (50 m × 0.25 mm i.d., 0.19 µm film thickness) coated with CP‐Sil 88 (Chrompack), with a temperature gradient from 140 to 220°C. Helium was used as a carrier gas at a constant pressure of 100 kPa.

Fatty acids were identified by comparison of retention times with those of a standard mixture (Supelco 37 FAME Mix, Sigma‐Aldrich, St. Louis, MO, USA), and results were expressed as relative percentages of total fatty acids based on peak area normalization, in accordance with international chromatographic standards.

### 2.3 Animal Study

To determine the effects of coffee beverage consumption (whole vs. decaffeinated) on normal and hyperlipidemic rats, 30 male Wistar rats weighing 350 ± 50 g were obtained from the animal house of the Federal University of Lavras (UFLA, Lavras, MG, Brazil). The animals were maintained at 23°C throughout the experimental period, under a 12 h light/dark cycle, with free access to commercial feed and water (ad libitum). The animal study was conducted using a completely randomized design.

The study was approved by the Ethics Committee on the Use of Animals (CEUA) of the UFLA under protocol no. 046/09, in accordance with Brazilian national legislation regulated by the National Council for the Control of Animal Experimentation (CONCEA).

#### 2.3.1 Preparation of the Hypercholesterolemic Diet

Hypercholesterolemia was induced using commercial chow (Nuvilab CR‐1, Nuvital Nutrientes S/A, Colombo, PR, Brazil) supplemented with cholesterol (0.5%) and cholic acid (0.25%), similar to previously reported diet‐induced hypercholesterolemia models in rats (Chiang et al. 1998). The feed was ground in an industrial blender, fortified, moistened with water, pelletized, and dried in a ventilated oven at 35°C for 48 h until complete drying. Animals in the negative control group (non‐hyperlipidemic) received the same commercial chow without cholesterol or cholic acid supplementation.

#### 2.3.2 Experimental Groups

The rats were randomly allocated into six experimental groups (*n* = 5 per group): C− (standard diet and water), C+ (hypercholesterolemic diet and water), WA (hypercholesterolemic diet and whole *Coffea arabica* beverage), DA (hypercholesterolemic diet and decaffeinated *C. arabica* beverage), WC (hypercholesterolemic diet and whole *C. canephora* beverage), and DC (hypercholesterolemic diet and decaffeinated *C. canephora* beverage). Coffee beverages were freshly prepared daily and administered via oral gavage at a dose of 7.2 mL/kg body weight for 42 consecutive days. This dose was based on the protocol described by Silvério et al. (2013) and was considered equivalent to a daily human consumption of approximately 400 mL of a filtered coffee beverage. At the end of the experimental period, the animals were fasted for 12 h and then anesthetized with thiopental (35 mg/kg). Blood was collected by cardiac puncture, and the liver was excised.

#### 2.3.3 Biological Sample Preparation

Blood samples were collected in siliconized tubes, either without additives or containing EDTA, and centrifuged at 1,500 × g for 10 min at room temperature to obtain serum and plasma. Plasma samples collected with EDTA were used for malondialdehyde (MDA) determination, whereas serum samples were used to assess the activities of superoxide dismutase (SOD) and glutathione peroxidase (GPx). An aliquot of serum was stored at −20°C for lipid profile analysis, and the remaining serum and plasma samples were stored in liquid nitrogen until analysis.

Liver samples were divided into four aliquots. One aliquot was homogenized (4:1, buffer:tissue, w/w) in phosphate buffer containing phenylmethylsulfonyl fluoride (PMSF, 50 µg/mL), benzamidine (100 µM), and leupeptin (50 µM), using a Potter tissue homogenizer under ice cooling. This homogenate was used for MDA determination. A second aliquot was homogenized at 4°C in Tris–HCl buffer (30 mM, pH 7.4) containing EDTA (5 mM), KCl (30 mM), sucrose (250 mM), PMSF (50 µg/mL), benzamidine (100 µM), and leupeptin (50 µM) and used for SOD and GPx activity assays. Homogenates were centrifuged at 600 × g for 10 min at 4°C. The remaining liver aliquots were used for total lipid determination and histopathological analysis.

Protein concentrations in blood and liver samples were determined using the Bradford assay (Bradford 1976).

#### 2.3.4 Determination of Triacylglycerols, Total Serum Cholesterol, HDL‐C, and Non‐HDL‐C Cholesterol

Commercial enzymatic colorimetric kits (Labtest, Lagoa Santa, MG, Brazil) were used to determine serum triacylglycerols, total cholesterol, and HDL‐C. Non‐HDL‐C was calculated as the difference between total cholesterol and HDL‐C concentrations.

#### 2.3.5 Determination of SOD and GPx Activities

Superoxide dismutase (SOD) activity was determined according to Oyanagui (1984). Serum and liver homogenate aliquots were incubated with hydroxylamine (0.1 M), hypoxanthine (0.01 M), and xanthine oxidase (4 × 10^−^^6^ U/mL) at 37°C for 30 min in the dark. Subsequently, sulfanilic acid, α‐naphthylenediamine, and glacial acetic acid were added and incubated at room temperature for 20 min. One unit of enzyme activity was defined as the amount producing 50% inhibition of the reaction. Glutathione peroxidase (GPx) activity was determined according to Sinet et al. (1975). Serum and liver homogenate aliquots were incubated with reduced glutathione (6 mM), phosphate buffer (50 mM, pH 7.0), NADPH (0.001 mM), and glutathione reductase (1 IU) for 3 min at 37°C. After incubation, t‐butyl hydroperoxide (0.07 g/mL) was added, and NADPH oxidation was monitored at 340 nm for 5 min. Results were expressed as units per mg of protein (U/mg protein).

#### 2.3.6 Lipid Peroxidation Assessment

Malondialdehyde (MDA) levels in plasma and liver homogenate samples were estimated by determining thiobarbituric acid reactive substances (TBARS) by high‐performance liquid chromatography (HPLC), according to the methodology described by Punchard and Kelly (1996).

The reaction of MDA with thiobarbituric acid was carried out using 50 µL of sample or standard, 250 µL of phosphoric acid, 450 µL of Milli‐Q water (Millipore, Billerica, MA, USA), and 250 µL of thiobarbituric acid, followed by incubation in a water bath at 95°C for 1 h. The solution was then cooled to 4°C, and 200 µL of the reaction mixture was combined with 360 µL of methanol and 40 µL of sodium hydroxide. This solution was filtered through a 0.45 µm membrane filter (Millipore) prior to chromatographic analysis.

MDA was determined by HPLC using a C18 column (4.6 mm × 25 cm) under reverse‐phase conditions, with fluorescence detection at 532 nm (excitation) and 553 nm (emission). Elution was isocratic, at a flow rate of 0.8 mL/min, using methanol/phosphate buffer (25 mM, pH 6.5) (50:50, v/v) as the mobile phase. Quantification was performed by external calibration with 1,1,3,3‐tetramethoxypropane, based on peak area integration (Punchard and Kelly 1996).

#### 2.3.7 Determination of Lipids in the Liver

Total liver lipids were determined according to the AOAC (2000) methodology. Liver samples were freeze‐dried in a freeze‐dryer (Liobrás, São Carlos, SP, Brazil; model L101) and ground in a mortar. Two grams of the resulting powder were weighed, placed in cellulose cartridges, and subjected to lipid extraction with petroleum ether for 6 h in a Soxhlet apparatus. The method is gravimetric, based on the mass difference before and after extraction, corresponding to the amount of material solubilized by the solvent. Results were expressed as percentage of total lipids relative to fresh liver weight.

#### 2.3.8 Histological Analysis of the Livers

Liver samples were fixed in 10% formalin for 24 h, after which the organs were sectioned, dehydrated, and cleared through a graded series of ethanol solutions, remaining for 30 min in each solution. Tissue processing was performed using three successive paraffin baths, each lasting 30 min, followed by embedding in paraffin blocks. The blocks were sectioned using a microtome to a thickness of 4–6 µm. The sections were mounted on glass slides and deparaffinized in alcoholic solutions. Slides were examined under a light microscope at 40× magnification. Histological alterations were classified as absent (−), mild (+), moderate (++), or severe (+++).

### 2.4 Statistical Analysis

For the characterization of bioactive compounds, four types of beverages were evaluated (whole arabica, decaffeinated arabica, whole canephora, and decaffeinated canephora), with five replicates per treatment. The biological assay followed a factorial design involving two coffee species (arabica and canephora), two processing conditions (whole and decaffeinated), and two diets (standard and hypercholesterolemic), with five replicates per treatment. Data were subjected to analysis of variance (ANOVA), and mean comparisons were performed using the Scott–Knott test at a 5% significance level (*p* < 0.05). Statistical analyses were conducted using SISVAR software, version 5.6 (Lavras, MG, Brazil) (Ferreira 2011).

## 3 Results and Discussion

### 3.1 Beverages Composition

Table 1 shows the average concentrations of the analyzed compounds. The highest caffeine concentrations were observed in the whole canephora samples, followed by whole arabica, as expected (Pietsch 2017). Decaffeinated beverages showed caffeine contents within the limits permitted by Brazilian legislation (ANVISA 2005), corresponding to a percentage reduction of 94% and 97% for arabica and canephora coffee beverages, respectively.

**TABLE 1:** Bioactive compounds in whole arabica (WA), decaffeinated arabica (DA), whole canephora (WC), and decaffeinated canephora (DC) coffee beverages.

	WA	DA	WC	DC
Caffeine (mg/L)	760 ± 10^B^	50 ± 10^C^	1,110 ± 50^A^	30 ± 10^C^
Total phenolics (g eq. gallic acid/L)	6.55 ± 0.040^C^	6.89 ± 0.020^B^	6.93 ± 0.002^B^	7.46 ± 0.03^A^
Total chlorogenic acids (mg/L)	422 ± 4^C^	748 ± 5^B^	352 ± 7^D^	1,268 ± 11^A^
3‐CQA (mg/L)	87 ± 1^C^	152 ± 2^B^	67 ± 2^C^	258 ± 15^A^
4‐CQA (mg/L)	136 ± 6^C^	237 ± 6^B^	138 ± 7^C^	420 ± 14^A^
5‐CQA (mg/L)	199 ± 5^C^	359 ± 2^B^	147 ± 2^D^	590 ± 15^A^
Trigonelline (mg/L)	170 ± 10^B^	360 ± 10^A^	90 ± 3^C^	380 ± 20^A^
Niacin (mg/L)	16 ± 1^A^	12 ± 1^B^	13 ± 2^B^	16 ± 1^A^
Total diterpenes (mg/L)	0.91 ± 0.06^B^	0.37 ± 0.07^D^	1.01 ± 0.09^A^	0.59 ± 0.03^C^
Kahweol (mg/L)	0.33 ± 0.03^A^	0.14 ± 0.05^B^	0.04 ± 0.004^C^	0.09 ± 0,0004^C^
Cafestol (mg/L)	0.58 ± 0.04^B^	0.23 ± 0.05^C^	0.97 ± 0.04^A^	0.50 ± 0,01^B^
Total tocopherols (µg/L)	20.94 ± 0.65^A^	15.13 ± 0.28^C^	18.79 ± 1.0^B^	14.01 ± 0.3^D^
α‐Tocopherol (µg/L)	10.31 ± 0.42^A^	7.73 ± 0.16^C^	8.01 ± 0.17^B^	6.56 ± 0.44^D^
β‐Tocopherol (µg/L)	10.63 ± 0.04^A^	7.40 ± 0.11^B^	10.79 ± 0.92^A^	7.46 ± 0.12^B^

Values are expressed as means ± standard deviation. Different capital letters within the same row indicate significant differences (p < 0.05) according to the Scott–Knott test.

The whole canephora coffee beverage (WC) also exhibited significantly higher concentrations of total phenolic compounds determined by the Folin–Ciocalteu method than the whole arabica coffee beverage (WA), as typically observed between these two species (Delorme et al. 2025). After decaffeination, the concentration of total phenolics increased in both beverages. The quantification of the individual chlorogenic acids (CGA) by HPLC, the major phenolic compounds in coffee, particularly 5‐CQA, 4‐CQA, and 3‐CQA (Park et al. 2024), was consistent, with the levels of CGA in the decaffeinated arabica and canephora coffee beverages being significantly higher than in their respective whole beverages, with the decaffeinated canephora coffee beverage (DC) standing out (Table 1).

Previous studies reported higher phenolic or CGA levels in decaffeinated coffees from different origins (Farah et al. 2006; Silvério et al. 2013; Park et al. 2024), and more recently, some decaffeinated blends showed higher total phenolic levels than traditional coffees (Costa et al. 2024). The increase observed in the present study may be related to multiple factors. Dichloromethane, although relatively selective for caffeine, can also promote the co‐extraction of other soluble constituents, thereby reducing bean mass and increasing the relative concentration of the remaining compounds (Toci et al. 2006). Increased bean porosity after decaffeination may also enhance extraction efficiency during beverage preparation (Pietsch 2017).

Furthermore, physical and chemical alterations induced by decaffeination increase the susceptibility of green beans to thermal effects during roasting, particularly hydrolysis and polymerization reactions (Shofinita et al. 2024; Lee 1999). These changes may allow shorter roasting times to achieve the same color, resulting in reduced roasting‐induced losses of chlorogenic acids in decaffeinated coffee. In contrast, Klikarová et al. (2022) observed a reduction in total CGA content in decaffeinated coffee samples, both roasted and unroasted, reporting that the content of the main isomer (5‐CQA) was reduced by 30–45%, while the 3‐CQA and 4‐CQA isomers remained practically unchanged, probably derived from the roasting degree of the samples analyzed, mostly under the medium‐dark range.

Knowing that chlorogenic acids contribute to the antioxidant activity of coffee beverages, their increase may be biologically relevant. However, very high levels can negatively affect sensory quality, since CGAs are among the compounds responsible for coffee bitterness (Park et al. 2024).

Trigonelline is a thermosensitive compound whose content may be indirectly influenced by decaffeination through changes in bean structure and roasting behavior. Nevertheless, during roasting, decaffeinated coffee exhibits lower losses of this compound compared to whole coffee (Park et al. 2024). In this study, roasted decaffeinated samples showed higher trigonelline contents than whole‐bean samples (Table 1). This effect may be explained by mechanisms similar to those proposed for CGA in decaffeinated samples. The shorter roasting time required for decaffeinated beans leads to reduced trigonelline degradation. Regarding species, the higher trigonelline concentrations observed in arabica coffees are consistent with previous reports (Perrone et al. 2008; Souza et al. 2010).

During roasting, trigonelline undergoes demethylation, leading to the formation of niacin (Jeszka‐Skowron et al. 2020), an essential vitamin for human metabolism, also known as nicotinic acid or vitamin B3. Lower trigonelline levels in decaffeinated samples may reduce niacin formation (Toci et al. 2006). In the present study, despite the higher trigonelline concentrations observed in decaffeinated coffees, a significant decrease in niacin levels was detected in the decaffeinated arabica samples, whereas a relative increase was observed in decaffeinated canephora samples (Table 1). These findings suggest that niacin formation is not solely dependent on residual trigonelline content but is also influenced by species‐specific matrix composition and roasting conditions.

Cafestol concentrations in whole coffee beverages (0.58 mg/L for WA and 0.97 mg/L for WC) were significantly higher than those in their decaffeinated counterparts (0.23 mg/L for DA and 0.50 mg/L for DC). However, for kaweol, this trend was observed only in arabica coffee, since decaffeinated canephora coffee exhibited higher kaweol levels than its whole‐bean counterpart (0.09 mg/L and 0.04 mg/L, respectively). The presence of kaweol in canephora coffee remains controversial, with some studies reporting its absence and others describing very low concentrations or trace levels (Souza et al. 2010). Given the low concentrations detected in filtered coffee beverages, it is more appropriate to discuss total diterpenes. Decaffeination led to a reduction in total diterpene content (Table 1), with decreases of 59% in DA beverages and 32% in DC beverages. This reduction may be attributed to the solubility of these lipidic compounds in dichloromethane, resulting in losses during the decaffeination process.

Very low levels of α‐ and β‐tocopherols were detected, likely due to the filter coffee preparation method, in which water passes through the coffee bed under minimal pressure, limiting the extraction of lipid‐soluble compounds. Alves et al. (2010) reported that filter‐prepared coffee beverages exhibited the lowest tocopherol concentrations among different brewing methods, with values comparable to those observed in the present study. In addition, decaffeinated beverages showed lower total tocopherol concentrations than their whole‐bean counterparts (Table 1). This finding is consistent with the reductions observed for diterpenes and supports the hypothesis that dichloromethane decaffeination may partially remove lipophilic constituents in addition to caffeine (Toci et al. 2006). Although tocopherols are susceptible to heat and oxidation, the lower levels observed in decaffeinated beverages are more likely associated with changes in the lipid fraction caused by the decaffeination process. Despite their low concentrations, tocopherols contribute to the overall antioxidant capacity of coffee beverages, as their lipophilic nature allows them to exert antioxidant effects in compartments distinct from those targeted by hydrophilic antioxidants (Alves et al. 2010).

Eight fatty acids were identified in the lipids of coffee beverages, and the average percentage and total fatty acid content are shown in Table 2. With a reduced lipid content (10–12 mg/L), the main fatty acids were linoleic and palmitic, followed by stearic and oleic acids, in agreement with other studies (Dong et al. 2017; Romano et al. 2014). The other fatty acids each represented less than 3.5% of the total fatty acids. Although Arabica coffee beans are known to contain higher amounts of extractable lipids (not evaluated in this study), no differences were observed between whole beverages. The same applies to decaffeinated beverages, even though dichloromethane is expected to extract lipids (Toci et al. 2006). The absence of significant differences in total fatty acids, particularly in canephora beverages, suggests that the impact of decaffeination on the lipid fraction may vary according to the class of compounds evaluated, with diterpenes and tocopherols appearing to be more affected than fatty acids under the conditions studied.

**TABLE 2:** Fatty acid composition (%) and total fatty acid (TFA, mg/L) in whole arabica (WA), decaffeinated arabica (DA), whole canephora (WC), and decaffeinated canephora (DC) coffee beverages.

Fatty acids		Beverages			
		WA	DA	WC	DC
C16:0	Palmitic	35.2 ± 1.6	35.6 ± 0.8	33.0 ± 0.2	31.2 ± 0.4
C18:0	Stearic	11.9 ± 0.3	11.0 ± 0.2	11.9 ± 0.2	11.6 ± 0.2
C18:1	Oleic	11.8 ± 2.7	10.3 ± 1.9	11.0 ± 0.1	9.9 ± 0.3
C18:2	Linoleic	35.0 ± 2.0	36.0 ± 0.7	38.0 ± 0.2	41.2 ± 0.3
C20:0	Arachidic	3.0 ± 0.4	2.5 ± 0.4	3.2 ± 1.0	3.0 ± 0.3
C18:3	α‐Linolenic	1.3 ± 0.3	2.6 ± 0.2	0.6 ± 0.1	1.2 ± 0.2
C22:0	Behenic	0.9 ± 0.1	0.8 ± 0.1	1.3 ± 0.1	0.9 ± 0.1
C24:0	Lignoceric	0.5 ± 0.1	1.1 ± 0.1	1.1 ± 0.9	1.0 ± 0.1
**Total fatty acid** (TFA, mg/L)		**11.90 ± 0.2^A^**	**9.82 ± 0.3^B^**	**11.94 ± 0.1^A^**	**12.04 ± 0.1^A^**

Values are expressed as means ± standard deviation. Different capital letters in the last row indicate significant differences (p < 0.05) according to the Scott–Knott test.

### 3.2 Animal Study

#### 3.2.1 Lipid Profile

Serum triacylglycerols, total cholesterol, HDL cholesterol (HDL‐c), and non‐HDL cholesterol are shown in Table 3. Triacylglycerols, total cholesterol, and non‐HDL cholesterol were significantly higher in the positive control (C+) (80 ± 8.8, 108 ± 7.1, and 94 ± 7.9 mg/dL, respectively) than in the negative control (C−) (45 ± 4.2, 57 ± 7.4, and 43 ± 6.8 mg/dL, respectively), confirming effective induction of hyperlipidemia, while HDL‐C did not differ between controls.

**TABLE 3:** Serum lipid profile in control groups (C− and C+) and rats treated with whole arabica (WA), decaffeinated arabica (DA), whole canephora (WC), and decaffeinated canephora (DC) coffee beverages.

Parameter (mg/dL)	C−	C+	WA	DA	WC	DC
Triacylglycerols	45 ± 4.2^B^	80 ± 8.8^A^	41 ± 2.9^B^	45 ± 6.0^B^	45 ± 5.0^B^	47 ± 6.4^B^
Total cholesterol	57 ± 7.4^C^	108 ± 7.1^A^	87 ± 13.5^B^	80 ± 13.1^B^	91 ± 8.3^B^	83 ± 10.4^B^
Non‐HDL cholesterol	43 ± 6.8^C^	94 ± 7.9^A^	74 ± 12.9^B^	65 ± 12.6^B^	78 ± 8.9^B^	70 ± 11.0^B^
HDL‐cholesterol	14 ± 1.4^A^	14 ± 0.9^A^	14 ± 2.1^A^	14 ± 1.5^A^	13 ± 1.5^A^	13 ± 1.7^A^

Values are expressed as means ± standard deviation. Different capital letters within the same row indicate significant differences (p < 0.05) according to the Scott–Knott test.

Triacylglycerol concentrations decreased from 80 ± 8.8 mg/dL in the C+ group to 41 ± 2.9, 45 ± 6.0, 45 ± 5.0, and 47 ± 6.4 mg/dL in the WA, DA, WC, and DC groups, respectively (48.8%, 43.8%, 43.8%, and 41.3% reductions). Non‐HDL cholesterol decreased from 94 ± 7.9 mg/dL to 74 ± 12.9, 65 ± 12.6, 78 ± 8.9, and 70 ± 11.0 mg/dL (21.3%, 30.9%, 17.0%, and 25.5% reductions), while total cholesterol decreased from 108 ± 7.1 mg/dL to 87 ± 13.5, 80 ± 13.1, 91 ± 8.3, and 83 ± 10.4 mg/dL (19.4%, 25.9%, 15.7%, and 23.1% reductions) in the WA, DA, WC, and DC groups, respectively. HDL‐cholesterol did not differ among the experimental groups. No significant differences were observed among coffee species or between whole and decaffeinated beverages (Table 3).

Experimental studies show that coffee can reduce triglycerides, LDL‐C and total cholesterol in obese or high‐fat diet rats (El‐Kersh et al. 2023; Feyisa et al. 2019), though effects on HDL‐c and in pharmacological hyperlipidemia models vary (Cavalcanti et al. 2022; Silvério et al. 2013), highlighting the influence of the experimental model.

The hypolipidemic effects of coffee have been attributed to the combined action of its bioactive compounds. Proposed mechanisms include modulation of digestion, intestinal absorption, and lipid transport, as well as changes in fatty acid β‐oxidation, lipolysis, and lipogenesis (Al‐Fawaeir et al. 2023; Farias‐Pereira et al. 2019; Vitaglione et al. 2019). Caffeine may inhibit systemic enzymes and modulate lipid metabolism, partly through antagonism of adenosine receptors and increased cyclic AMP (cAMP) generation (Arroyave‐Ospina et al. 2023). Increased cAMP levels and sympathetic activation can stimulate catecholamine release and β‐adrenergic receptor activation, thereby promoting lipolysis and the hydrolysis of triglycerides in adipose tissue (Barcelos et al. 2020; Panchal et al. 2012).

Beyond caffeine, phenolics and melanoidins contribute to coffee's metabolic benefits (Vitaglione et al. 2019; Xiao et al. 2014). Chlorogenic acids (CGAs) play a central role, exhibiting anti‐inflammatory and antioxidant activities and reducing triglycerides, cholesterol, LDL‐c oxidation, and bile acid synthesis (Colombo and Papetti 2021). Trigonelline and cafestol may downregulate lipogenic enzymes, while other phenolic acids stimulate HDL formation and cholesterol efflux (Farias‐Pereira et al. 2019; Cavalcanti et al. 2022). These findings support a hypolipidemic effect independent of caffeine.

#### 3.2.2 Serum SOD and GPx Enzyme Activity

**FIGURE 1:**
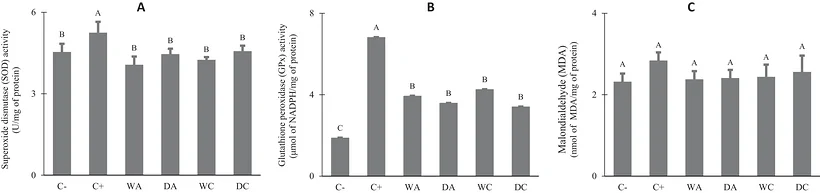
Antioxidant enzyme activity (A, B) and lipid peroxidation (C) in blood of control groups (C− and C+) and rats treated with whole arabica (WA), decaffeinated arabica (DA), whole canephora (WC), and decaffeinated canephora (DC) coffee beverages. (A) Superoxide dismutase, (B) glutathione peroxidase, and (C) malondialdehyde. Values are expressed as means ± standard deviation. Different letters indicate significant differences among groups (*p* < 0.05) according to the Scott–Knott test.

The hypercholesterolemic diet significantly increased serum SOD activity from 4.54 ± 0.40 to 5.25 ± 0.43 U/mg of protein in C+ compared to C− (15.6% increase) and serum GPx activity from 1.90 ± 0.80 to 6.83 ± 1.36 µmol of NADPH/mg of protein (259.0% increase). Coffee‐treated animals showed serum SOD activity ranging from 4.07 ± 0.38 to 4.57 ± 0.29 U/mg of protein and serum GPx activity ranging from 3.42 ± 0.94 to 4.27 ± 0.53 µmol of NADPH/mg of protein, values comparable to C− and significantly lower than C+, with no differences among coffee groups (Figure 1).

SOD catalyzes the dismutation of superoxide into hydrogen peroxide (H_2_O_2_) and oxygen (O_2_), which is subsequently eliminated by GPx and other antioxidant enzymes to prevent the formation of hydroxyl radicals (Ighodaro and Akinloye 2018).

Only enzyme activity was measured, but increased expression may contribute to higher activity in C+. ROS exposure activates the Nrf2–ARE pathway, inducing SOD, catalase, GPx, glutathione, peroxiredoxins, NADPH, and other components of the antioxidant defense system (Li et al. 2023b; Ighodaro and Akinloye 2018).

Hypercholesterolemic diet increased serum SOD and GPx, likely due to enhanced ROS activating Nrf2–ARE. Coffee treatment prevented this increase, suggesting that antioxidant and hypolipidemic compounds (e.g., phenolics and niacin) reduce serum lipids and ROS, attenuating pro‐oxidant events and maintaining redox homeostasis (El‐Kersh et al. 2023).

Overall, coffee beverages, irrespective of species or decaffeination, effectively controlled systemic oxidative stress in hyperlipidemic animals. In agreement, Costa et al. (2024) showed that decaffeination did not reduce antioxidant activity in Cerrado Mineiro coffee blends, reinforcing that this potential is mainly due to non‐caffeine bioactives (phenolics and melanoidins).

#### 3.2.3 Lipid Peroxidation in Plasma

Mean plasma levels of the lipid peroxidation marker malondialdehyde (MDA) are shown in Figure 1C, with no significant differences among groups. MDA concentrations were 2.316 ± 0.222 nmol MDA/mg of protein in the C− group and 2.836 ± 0.207 nmol MDA/mg of protein in the C+ group. In coffee‐treated animals, MDA levels ranged from 2.382 ± 0.223 to 2.566 ± 0.373 nmol MDA/mg of protein. Despite the increase in SOD and GPx activity observed in the positive control group, MDA concentrations remained similar across groups. Since SOD and GPx constitute important components of the endogenous antioxidant defense system, the increase in their activities may represent an adaptive response to the oxidative challenge imposed by the hypercholesterolemic diet. The absence of differences in MDA concentrations suggests that lipid peroxidation remained controlled under the experimental conditions. Similarly, coffee‐treated animals exhibited MDA levels comparable to those of both control groups, indicating that neither coffee species nor decaffeination status adversely affected oxidative status (Taslim et al. 2023; Zamora et al. 2023)..

#### 3.2.4 Lipid Content in the Liver

The liver weight‐to‐body weight ratio was significantly higher in C+ than in C− (0.04 ± 0.001 vs. 0.02 ± 0.003), indicating that the hypercholesterolemic diet induced hepatomegaly. All coffee beverages mitigated liver weight gain, with values of 0.03 ± 0.001–0.03 ± 0.002 (25.0% reduction vs. C+). Similarly, hepatic lipid content was elevated in C+ (10.8 ± 0.8% vs. 1.6 ± 0.8% in C−), reflecting lipid accumulation driven by cholesterol and cholic acid, which enhances absorption and inhibits conversion to bile acids, promoting steatosis (Chun et al. 2022). Coffee‐treated animals showed reduced hepatic lipid content (7.9 ± 1.1–8.4 ± 0.3%) compared to C+ (25.0% reduction), with no differences among coffee types, although levels remained higher than in C− (Table 4).

**TABLE 4:** Hepatic lipid content and liver weight‐to‐body weight ratio in control groups (C− and C+) and rats treated with whole arabica (WA), decaffeinated arabica (DA), whole canephora (WC), and decaffeinated canephora (DC) coffee beverages.

	C−	C+	WA	DA	WC	DC
**Total lipids (%, w/w)**	1.6 ± 0.8^C^	10.8 ± 0.8^A^	7.9 ± 1.1^B^	8.0 ± 0.9^B^	8.1 ± 0.7^B^	8.4 ± 0.3^B^
**Liver‐to‐body weight ratio**	0.02 ± 0.003^C^	0.04 ± 0.001^A^	0.03 ± 0.002^B^	0.03 ± 0.001^B^	0.03 ± 0.001^B^	0.03 ± 0.001^B^

Values are expressed as means ± standard deviation. Different capital letters within the same row indicate significant differences (p < 0.05) according to the Scott–Knott test.

Several studies confirm that both whole and decaffeinated coffee reduce hepatic fat deposition (Brandt et al. 2019; Sinha et al. 2014; Vitaglione et al. 2019; Zhang et al. 2015; Lima et al. 2013). Mechanisms include inhibition of lipogenesis, stimulation of fatty acid oxidation, downregulation of SREBP‐1c, upregulation of PPAR‐α and acyl‐CoA oxidase 1, and reduced intestinal fatty acid absorption via decreased FAT/CD36 expression (Alegret et al. 2025; Vitaglione et al. 2019). Coffee may also reduce circulating cholesterol through enhanced LXR‐α–mediated efflux, attenuate oxidative stress via Nrf2 activation, and improve gut barrier function, collectively protecting against diet‐induced hepatic steatosis independent of caffeine content (Brandt et al. 2019; Vitaglione et al. 2019).

#### 3.2.5 SOD and GPx Enzyme Activity in the Liver

**FIGURE 2:**
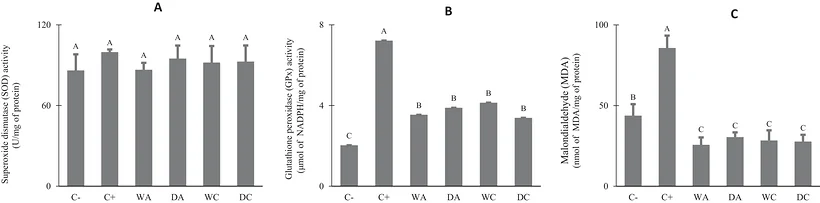
Antioxidant enzyme activity (A, B) and lipid peroxidation (C) in liver control groups (C− and C+) and rats treated with whole arabica (WA), decaffeinated arabica (DA), whole canephora (WC), and decaffeinated canephora (DC) coffee beverages. (A) Superoxide dismutase, (B) glutathione peroxidase, and (C) malondialdehyde. Values are expressed as means ± standard deviation. Different letters indicate significant differences among groups (*p* < 0.05) according to the Scott–Knott test.

Figures 2A and 2B show that, unlike serum, hepatic SOD activity did not differ among groups (C−: 66.9 ± 12.1 U/mg of protein; C+: 98.8 ± 7.1 U/mg of protein; coffee‐treated groups: 75.6–108.0 U/mg of protein). In contrast, hepatic GPx activity was significantly higher in the C+ group (7.224 ± 1.205 µmol of NADPH/mg of protein) than in the C− group (2.038 ± 0.179 µmol of NADPH/mg of protein). No significant differences were observed among coffee‐treated groups, whose GPx activities ranged from 3.538 ± 1.148 to 4.146 ± 0.773 µmol of NADPH/mg of protein; however, all exhibited significantly lower activity than the C+ group.

Superoxide anion can react with nitric oxide to form peroxynitrite, reducing SOD substrate availability, while hydroperoxide accumulation may stimulate GPx activity, explaining the observed pattern (Halliwell and Gutteridge 2007; Ighodaro and Akinloye 2018). Coffee bioactive compounds may enhance non‐enzymatic antioxidant defenses, reducing the need for enzymatic activity, whereas hypercholesterolemic diet‐induced lipid oxidative damage promotes oxidative stress.

Phenolic acids and other dietary antioxidants can act systemically, modulating enzyme activity or scavenging reactive oxygen species to preserve endogenous defenses, though mechanisms remain incompletely understood (Batista et al. 2023; Soares et al. 2021; Ramos et al. 2022). Intracellular isoforms of SOD and GPx differ from extracellular forms, and organ‐specific variations in oxidant/antioxidant balance may explain differences in antioxidant responses to ROS.

#### 3.2.6 Lipid Peroxidation in the Liver

Animals in C+ showed significantly higher hepatic MDA than C− (85.72 ± 7.74 vs. 43.8 ± 7.05 nmol MDA/mg of protein), indicating that increased GPx activity alone was insufficient to prevent lipid peroxidation. Coffee‐treated hyperlipidemic animals exhibited reduced hepatic MDA (25.63 ± 4.60–30.48 ± 2.94 nmol MDA/mg of protein) compared to C+ (67.2–70.1% reduction), with levels also lower than C− (Figure 2C), suggesting a synergistic effect of GPx and coffee‐derived antioxidants. Coffee also reduced hepatic lipid accumulation (Table 4), likely decreasing susceptibility to peroxidation. No differences were observed due to species or decaffeination. Overall, 42‐day coffee consumption effectively reduced hepatic lipid peroxidation, supporting protection of cell membranes against oxidative damage.

Similar studies confirm coffee's antioxidant effects. CGAs, among the most abundant polyphenols, are absorbed and exert antioxidant and anti‐inflammatory activity in tissues (Bao et al. 2018; Soares et al. 2021) and can inhibit LDL‐c oxidation (Bittar et al. 2024). Maillard reaction products from roasting also contribute to antioxidant activity, with darker‐roasted coffee associated with lower lipid peroxidation (Anese et al. 2023). Thus, the protective effects observed likely result from multiple coffee‐derived compounds, independent of caffeine content.

#### 3.2.7 Histological Analysis

**FIGURE 3:**
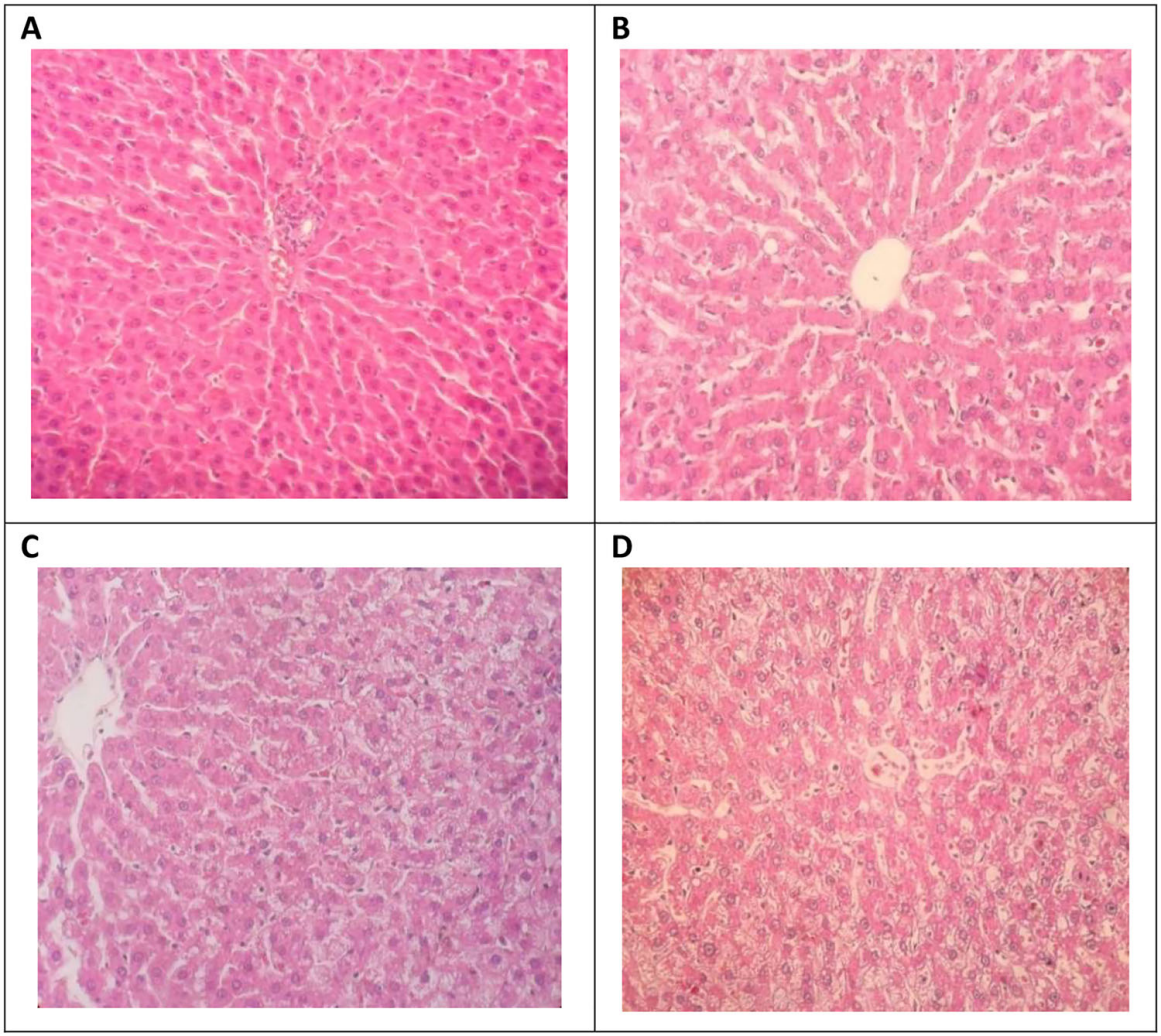
Representative photomicrographs of rat liver and classification of histological alterations: (A) normal; (B) mild lesion (+); (C) moderate lesion (++); and (D) severe lesion (+++). Hematoxylin and eosin (H&E) staining, 40× magnification.

Hepatic steatosis, characterized by intracellular fat accumulation due to increased synthesis or impaired utilization, transport, or excretion, can lead to metabolic dysfunction, inflammation, and advanced liver diseases (Ma et al. 2023). The C+ group exhibited severe steatosis, with numerous lipid vacuoles in periportal and midzonal regions, while the centrilobular region was mildly affected (Figure 3D). Coffee‐treated groups showed reduced liver injury compared to C+, reinforcing a hepatoprotective effect. The WA group had mostly mild lesions, DA mostly moderate lesions; WC showed 60% mild and 40% moderate lesions, and DC had 60% moderate, 30% mild, and 10% severe lesions.

Hepatoprotection was more pronounced in decaffeinated coffee for both species. Literature attributes coffee's benefits mainly to antioxidant compounds, though effects vary with species, chemical composition, processing, and preparation (Cavalcanti et al. 2022). Some studies report reduced antioxidant capacity after decaffeination based on in vitro antioxidant assays (Lima et al. 2010), whereas others found no difference between conventional and decaffeinated coffees using similar in vitro approaches (Moreira et al. 2005), suggesting that caffeine may not be the primary determinant of antioxidant activity. The relative contribution of phenolic compounds and Maillard reaction products remains unclear, but additive or synergistic interactions likely enhance antioxidant activity via radical stabilization mechanisms (Martins et al. 2023; Nicoli et al. 1997).

## 4 Conclusion

The results of this study indicate that decaffeination induces changes in the chemical composition of filtered coffee beverages, particularly a reduction in compounds from the lipid fraction, including total fatty acids, total tocopherols, and total diterpenes. In addition, higher concentrations of chlorogenic acids and trigonelline were observed in decaffeinated coffee samples.

In vivo, the hypolipidemic and antioxidant effects of the beverages in rats fed a hypercholesterolemic diet were not species‐ or decaffeination‐dependent. The only exception was the histopathological analysis, which suggested reduced liver protection in some decaffeinated and species‐dependent groups; however, it is important to note that histological evaluation is inherently subjective and depends on the observer's interpretation. Therefore, the present study demonstrates that caffeine removal does not reduce the antioxidant potential of coffee beverages, since decaffeinated coffees exhibited antioxidant and hypolipidemic activities comparable to those of their whole‐bean counterparts.

## Author Contributions

**Adriene Ribeiro Lima**: conceptualization, investigation, formal analysis, writing – review and editing, writing – original draft, visualization, methodology, data curation, validation. **Jeniffer Ferreira de Miranda**: writing – review and editing, visualization, data curation. **Juliana Mesquita Freire**: investigation. **Paula Kujbida**: writing – review and editing. **Sheila Andrade Abrahão**: investigation. **Eulália Mendes**: investigation. **José Avelino Alves Andrade da Silva**: investigation. **Fernanda Borges de Araújo Paula**: supervision, project administration, resources, formal analysis, methodology. **Stella Maris da Silveira Duarte**: conceptualization, supervision, resources, project administration, formal analysis, methodology. **Rosemary Gualberto Fonseca Alvarenga Pereira**: conceptualization, supervision, resources, funding acquisition, project administration, formal analysis. **Susana Casal**: supervision, resources, writing – review and editing, methodology, data curation, validation, formal analysis.

## Conflicts of Interest

The authors declare no conflicts of interest.
